# Childhood neurodevelopmental markers and risk of premature mortality: Follow-up to age 60–65 years in the Aberdeen Children of the 1950s study

**DOI:** 10.1371/journal.pone.0255649

**Published:** 2021-08-18

**Authors:** Adele Warrilow, Geoff Der, Sally-Ann Cooper, Helen Minnis, Jill P. Pell

**Affiliations:** 1 Institute of Health and Wellbeing, University of Glasgow, Glasgow, United Kingdom; 2 MRC/CSO Social and Public Health Sciences Unit, University of Glasgow, Glasgow, United Kingdom; Medical Research Council, SOUTH AFRICA

## Abstract

**Background:**

Individual neurodevelopmental disorders are associated with premature mortality. Little is known about the association between multiple neurodevelopmental markers and premature mortality at a population level. The ESSENCE (Early Symptomatic Syndromes Eliciting Neurodevelopmental Clinical Examinations) approach considers multiple neurodevelopmental parameters, assessing several markers in parallel that cluster, rather than considering individual diagnostic categories in isolation.

**Objectives:**

To determine whether childhood neurodevelopmental markers, including reduced intellectual functioning, are associated with all-cause premature mortality.

**Methods and procedures:**

In a general population cohort study (n = 12,150) with longitudinal follow up from childhood to middle age, Cox proportional hazard models were used to study the associations between childhood neurodevelopmental markers (Rutter B scale and IQ) and premature all-cause mortality.

**Outcomes and results:**

The cognitive measures and 21 of the 26 Rutter B items were significantly associated with premature mortality in bivariate analyses with hazard ratios from 1.24 (95% CI 1.05–1.47) to 2.25 (95% CI 1.78–2.90). In the final adjusted model, neurodevelopmental markers suggestive of several domains including hyperactivity, conduct problems and intellectual impairment were positively associated with premature mortality and improved prediction of premature mortality.

**Conclusions:**

A wide range of neurodevelopmental markers, including childhood IQ, were found to predict premature mortality in a large general population cohort with longitudinal follow up to 60–65 years of age.

**Implications:**

These findings highlight the importance of a holistic assessment of children with neurodevelopmental markers that addresses a range of neurodevelopmental conditions. Our findings could open the door to a shift in child public mental health focus, where multiple and/or cumulative markers of neurodevelopmental conditions alert clinicians to the need for early intervention. This could lead to a reduction in the risk of broad health outcomes at a population level.

## Introduction

Several studies have already demonstrated associations between individual neurodevelopmental disorders and premature mortality: Autism spectrum disorder (ASD) [1], Attention Deficit Hyperactivity Disorder (ADHD) [2], Conduct Disorder [3, 4], and Intellectual Disability [5] are all associated with an increased risk of premature death. Despite these disorders being conceptualised as separate entities, comorbidity is in fact “a rule rather than an exception” [6] and the broad range of symptoms of neurodevelopmental disorders are now known to have a common genetic underpinning [7]. Although there are some syndrome-specific genetic factors, they are of much smaller magnitude and unique environmental factors are largely what drives the manifestation of the underlying neurodevelopmental profile towards a more specific diagnostic category [7].

The concept of ESSENCE (Early Symptomatic Syndromes Eliciting Neurodevelopmental Clinical Examinations) was developed to take account of the clustering of neurodevelopmental disorders and the overlap in symptoms, even when they fall short of diagnostic thresholds [8]. The ESSENCE approach highlights that it is rare for one neurodevelopmental problem to occur in isolation, that individual symptoms are markers for the likely presence of additional neurodevelopmental problems, that markers of neurodevelopmental disorders can cross diagnostic boundaries, that clinical presentations can change throughout the life course and that “sub-clinical” markers across a range of diagnostic areas might be more important for predicting health outcomes than individual diagnoses. We use the term “markers” here because the term “symptoms” implies reference to a particular disorder whereas it has now been clearly shown that these markers often index more than one disorder [7]. There is still some debate about whether symptoms of certain childhood-onset disorders, particularly oppositional and conduct disorders, should be regarded alongside other Early Symptomatic Syndromes, but since both neurobiological and environmental risk factors are important in the development of conduct disorder as for all neurodevelopmental disorders, we regard it as logical to include markers such as aggression, rule breaking etc. In addition, common childhood symptoms such as anxiety and depression are extremely common in neurodevelopmental conditions so may be useful markers for these disorders [9]. Since the publication of the seminal ESSENCE paper in 2010, robust evidence has begun to accrue that, in population-based longitudinal research, childhood neurodevelopmental problems are best considered together [10, 11]. It has also become clear that children with “behaviour problems” are, in fact, likely to have neurodevelopmental problems underpinning these [12].

All neurodevelopmental disorders are associated with a wide range of physical and mental health problems across the lifespan. For example, nearly all major chronic medical conditions are significantly more common among adults with a health record of ASD compared to controls [13] and there are higher rates of long term medical conditions among both children and adults with ASD compared with the general population [14, 15]. Deceased adults with ASD were found to have had higher rates of most health problems compared with deceased community controls [16]. People with intellectual disabilities also have more health problems than other people [17]. Several prospective longitudinal studies have shown that childhood ADHD is associated with impaired general physical and mental health in adulthood [18–20] and ADHD symptoms persisting into early adulthood are associated with multi-morbidity [21]. Recent research has shown that tic disorders are associated with increased cardiovascular risk [22].

The risk of poor health outcomes, including premature mortality, is even greater if a child has more than one neurodevelopmental disorder. In a Swedish population-based cohort of 27,122 participants with ASD and 2,672,185 controls, ASD was associated with an increased risk of all-cause premature mortality and with most specific causes of premature mortality, particularly among those with co-existing intellectual disabilities [1]. In a Danish population cohort study, ASD plus one or more comorbid neurological, behavioural or mental disorders was associated with a higher risk of premature mortality [23]. In a Danish national register study of 1,922,248 individuals, including 32,061 with ADHD, ADHD was associated with increased mortality, with the mortality rate ratio more than doubling in the presence of comorbid behavioural disorders [2].

Until recently, the implications of the co-occurrence of more than one disorder has not been addressed in the scientific literature. It was only in the most recent edition of the Diagnostic Statistical Manual of Mental Disorders, 5^th^ edition (DSM-V) published in 2013 that the simultaneous diagnosis of ADHD and autism was formally recognised as possible (previously ASD was an exclusion criterion for the diagnosis of ADHD). This failure to take clustering of neurodevelopmental disorders into account has often led to the exclusion from studies of people with more than one diagnosis, yet clustering is extremely common: e.g. 95% of people with ASD have a co-existing disorder and more than 50% have more than four co-existing disorders [24]. Focussing on isolated disorders may well have led to an underestimation of the disease burden, and burden of premature mortality, associated with neurodevelopmental conditions across the lifespan. The recent findings that the full range of neurodevelopmental symptoms contribute to the common neurodevelopmental genetic factor [7] could have important implications for public health policy. The traditional clinical focus on those children who meet diagnostic criteria for a specific diagnosis may have led to missed opportunities for prevention in children who have the neurodevelopmental genetic profile yet are “sub-syndromal” for any particular disorder.

We therefore aim to address the question: Can a broad range of child neurodevelopmental markers predict premature mortality?

Despite rapidly accruing evidence that a multi-symptom approach to childhood neurodevelopment should underpin population research, no studies have thus far taken such an approach to examine premature mortality. Traditional registry studies are unable to address this because registers are kept according to diagnostic categories, which historically have been exclusionary. Participants in the newer large cohort studies, designed with this holistic approach in mind (e.g. [25, 26]), are not yet old enough to robustly examine premature mortality. Older UK cohorts include measures that capture the wide range of emotional and behavioural neurodevelopmental markers. Although these scales were traditionally used to give global scores for psychopathology or to indicate individual likely diagnoses, the individual items are available and relevant, giving us a unique opportunity to examine this important question.

## Materials and methods

### Ethical considerations

The use of an anonymised subset of cohort data was approved by the Children of the 1950s Study Steering Group (project manager contact details https://www.abdn.ac.uk/iahs/research/public-health-nutrition/profiles/h.clark). Permission to link to health data was granted by the Public Benefit and Privacy Panel for Health and Social Care (PBPP) (Application 1617–0225/Warrilow). Data was analysed within the Grampian Data Safe Haven.

### Participants

Participants were from the Aberdeen Children of the 1950s (ACoNF) study. Detailed descriptions of the cohort have been previously published [27, 28]. In brief, ACoNF is a general population cohort of children attending primary schools in Aberdeen in the 1960s, including specialist schools for children with learning disabilities. In March 1964, when participants were aged between eight and 14 years old, routinely conducted cognitive test scores were recorded as well as measures of reading ability and the Rutter B Scale, a teacher-reported measure of child neurodevelopmental markers. In 1999, the cohort, then aged between 43 and 49 years old, was traced via the NHS Central Registry to ascertain vital status and residency. Participants were flagged at the NHS Central Register for the ACoNF study to receive notification of future deaths and emigration. Surviving participants resident in the UK were followed up via a postal questionnaire in 2001–2003. In this study, participants comprised those who were traced and flagged for follow-up who also had complete childhood data.

### Child neurodevelopmental markers

The Rutter B scale is a 26-item questionnaire, completed for each pupil by the teacher that assesses a wide range of emotional and behavioural symptoms which commonly occur in several of the neurodevelopmental disorders, and which henceforth will be referred to as neurodevelopmental markers. Each question represents an individual neurodevelopmental marker and includes those markers typical of children with specific disorders: tic disorder (e.g. “has twitches, mannerisms or tics”); autism (e.g. “solitary”, “fussy”) and ADHD (e.g. “fidgety”, “poor concentration”)–although children with autism are often hyperactive and children with ADHD often have tics etc. The full wording of the scale items is listed in S1 Appendix alongside the shortened form used throughout the text. The questionnaire has been widely utilised within population research and has demonstrated good psychometric properties [29]. Re-test reliability was 0.89 for ratings by the same teacher two months apart and inter-rater reliability was 0.79 for ratings by separate teachers (last term in infant school versus first term in junior school) [30]. Validity has been assessed by comparison of children who were referred to psychiatric services to those who were not, comparison with psychiatric interview results and by comparison with other instruments e.g. the Child Behaviour Checklist [29, 30].

The 26 questions have three possible answers: “does not apply”, “applies somewhat” and “definitely applies”). We collapsed these to “applies” (1) or “not” (0).

### Cognitive measures

In the 1960s, children in Scotland routinely sat intelligence quotient (IQ) tests within six months of their seventh, ninth and eleventh birthdays. At age eleven years, the Moray House Verbal Reasoning Tests 1 and 2 were carried out. The mean of these was used as our measure of IQ. If only one test result was available this was used. If there was no age eleven test score available, the score from the test at age seven, the Moray House Picture Intelligence Test (No. 1 or 2), was used. If at age seven years children were thought not able to complete the standard test then an alternative IQ measure was used and if unable to complete any IQ test, as a result of low cognitive ability, a score of 50 was recorded. To account for any differences in scaling of these age-standardised measures, IQ was further standardised as a z-score and the inverse z-score used within the statistical modelling to aid interpretation.

Reading ability was assessed in December 1962 using the National Foundation for Educational Research (NFER) Sentence Reading Test 1 (for children aged between seven and nine years) or the NFER Reading Test N.S.6 (for children aged ten or 11 years old). The reading quotient (RQ) score was transformed into a z-score and the inverse used in the statistical modelling.

### Vital status

Vital status was ascertained from two sources: the UK NHS Central Register and the NHS Scotland Information Services Division. Information from the UK NHS Central Register was obtained as part of the ACoNF study data extraction (to June 2014) which includes all those deaths in the decades prior to the study participants being retraced/recontacted as well as setting up a system to record all deaths occurring thereafter. Information on deaths in Scotland to December 2015 was provided by the NHS Scotland Information Services Division eData Research and Innovation Service (eDRIS).

Permission was obtained from the Public Benefit and Privacy Panel for Health and Social Care (PBPP). To maintain confidentiality, dates of birth and death were provided as month and year only.

### Statistical analyses

Preliminary analyses employed Chi-square tests to examine the association of vital status with the presence or absence of each of the Rutter items and t-tests for the association with IQ and Reading Quotient.

Cox proportional hazards regression was used for the main analyses. Age at death or age at censor date was calculated in months. Censorship occurred at i) date of emigration from the UK, ii) June 2014 for participants traced and flagged by the NHS Central Register without a Scottish health record, or iii) December 2015 if participants were matched to a Scottish Health record at eDRIS.

Separate survival analysis models were carried out for each individual Rutter scale item, and for Reading Quotient and IQ. A selected multivariable model was derived using backward elimination from a model containing all Rutter items, RQ and IQ. Age was not associated with Rutter B items and gender only slightly (see S1 Table) so age and gender were not included in the model. Of the Rutter B items only one variable (attention) showed a significant interaction with gender (p = 0.014). All statistical analyses were carried out using STATA v13 within the Grampian Data Safe Haven.

## Results

Of the original 12,150 ACoNF study participants, 909 (7.5%) were excluded from the analyses: 89 because they were not able to be traced in 1999 and 820 because they had missing data on one or more childhood measures. The final study population therefore comprised 11,241 participants of whom 5,844 were male and 5,397 were female. Of the 11,241 participants, 1,167 (10.4%) were known to have died prematurely by the end of December 2015–63.7% of these deaths were in males and 36.3% in females. Table 1 shows the frequency of responses to the 26 individual Rutter B behaviours. A wide range of neurodevelopmental markers (18 out of the 26 Rutter B items) were more common in the group who experienced premature mortality. The mean Intelligence Quotient (IQ) and Reading Quotient (RQ) scores are presented in Table 2 and are significantly lower in those with premature mortality.

**Table 1:** Frequency of neurodevelopmental markers reported on Rutter B scale by vital status.

	Cohort (n = 11,241)		Died (n = 1,167)		Alive (n = 10,074)		P value (χ^2^, 1df)
	n	%	n	%	n	%	
restless	1,899	16.89	254	21.77	1,645	16.33	<0.001
truants	208	1.85	46	3.94	162	1.61	<0.001
fidgety	2,546	22.65	342	29.31	2,204	21.88	<0.001
destroys	362	3.22	69	5.91	293	2.91	<0.001
fights	1,240	11.03	184	15.77	1,056	10.48	<0.001
not liked	1,148	10.21	155	13.28	993	9.86	<0.001
worries	1,946	17.31	204	17.48	1,742	17.29	0.926
solitary	1,419	12.62	161	13.80	1,258	12.49	0.203
irritable	928	8.26	143	12.25	785	7.79	<0.001
unhappy	902	8.02	112	9.60	790	7.84	0.029
tics	417	3.71	54	4.63	363	3.60	0.141
sucks fingers	623	5.54	70	6.00	553	5.49	0.323
bites nails	2,227	19.80	272	23.31	1,955	19.41	0.001
school absences	613	5.45	98	8.40	515	5.11	<0.001
disobedient	1,266	11.26	203	17.40	1,063	10.55	<0.001
attention	3,380	30.07	465	39.85	2,915	28.94	<0.001
afraid of new things	1,686	15.00	186	15.94	1,500	14.89	0.462
fussy	661	5.88	58	4.97	603	5.99	0.355
lies	809	7.20	147	12.60	662	6.57	<0.001
steals	233	2.07	52	4.46	181	1.80	<0.001
wets or soils	153	1.36	14	1.20	139	1.38	0.821
aches and pains	394	3.51	58	4.97	336	3.34	0.001
tears/school refusal	61	0.54	4	0.34	57	0.57	0.674
stutters	237	2.12	34	2.91	203	2.02	0.035
other speech problem	464	4.13	72	6.17	392	3.89	<0.001
bullies	558	4.96	84	7.20	474	4.71	<0.001

n: number; χ^2^: Pearson Chi-square test; df: degrees of freedom.

**Table 2:** Mean cognitive and behavioural scores.

	Overall Mean (SD)	Died n (%)	Alive n (%)	P (t test)
IQ	104.00 (13.84)	100.24 (14.11)	104.43 (13.74)	p<0.001
RQ	99.02 (15.08)	95.34 (15.74)	99.45 (14.94)	p<0.001

IQ: intelligence quotient, RQ: reading quotient, SD: standard deviation, n: number.

**Fig 1:**
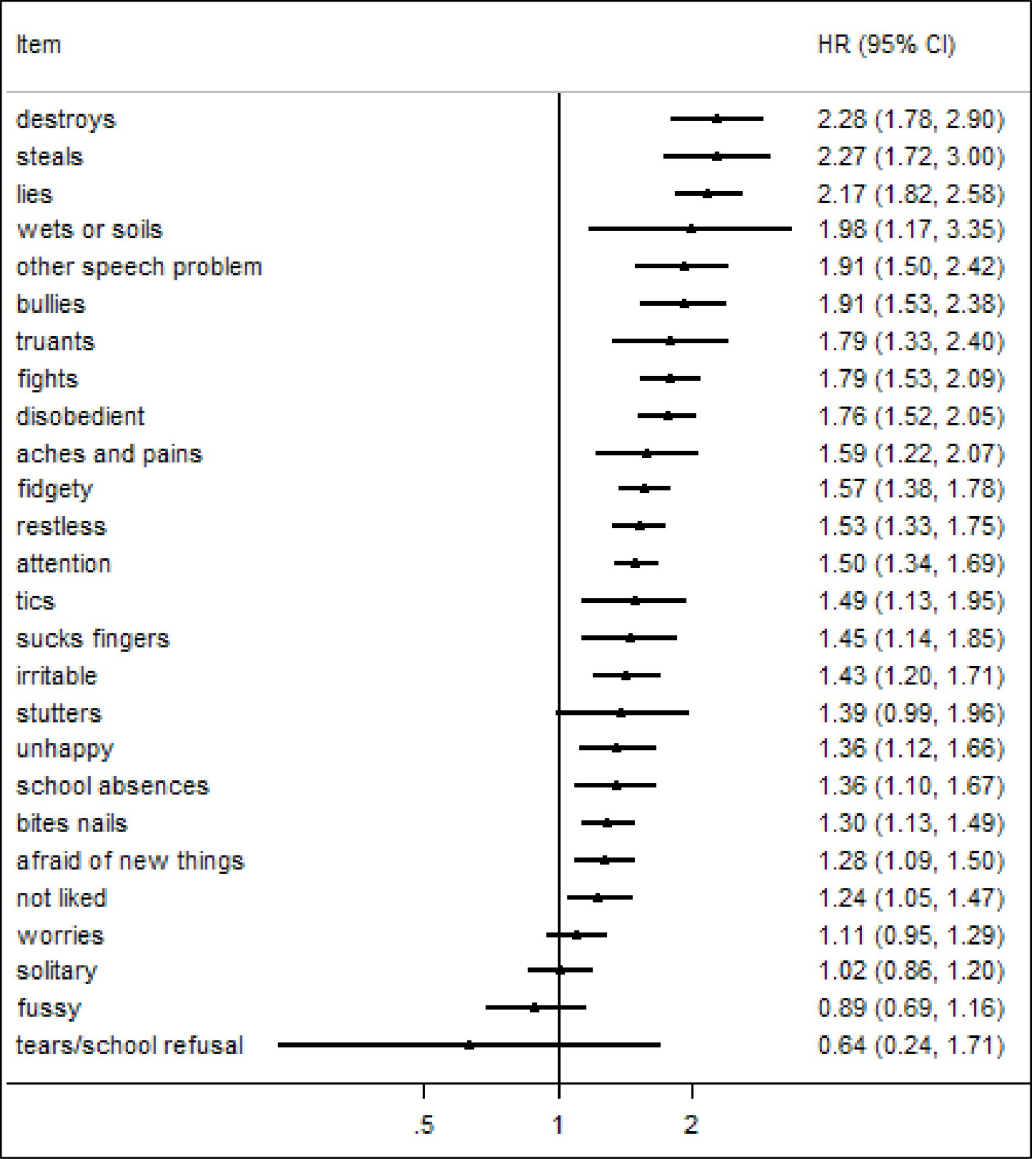
Mortality risk associated with each of the Rutter B items (n = 11,241).

The hazard ratios derived from the univariate Cox proportional hazard analyses are shown in Fig 1. There was a significant positive association with premature mortality for 21 of the 26 individual Rutter B behaviour items with hazard ratios ranging from 1.24 (CI: 1.05, 1.47) to 2.28 (CI: 1.78, 2.90). Cognitive measures also had a significant association in univariate analyses–for a one standard deviation decrease in IQ there was a 29% greater risk of death, and for RQ a 16% greater risk of death per standard deviation decrease (IQ (inverse standardised) HR: 1.29, 95% CI: 1.22, 1.36, p<0.001; RQ (inverse standardised) HR: 1.16, 1.10,1.23, p<0.001). Adjusting for gender made only very small differences to hazard ratios–see S1 Table.

**Fig 2:**
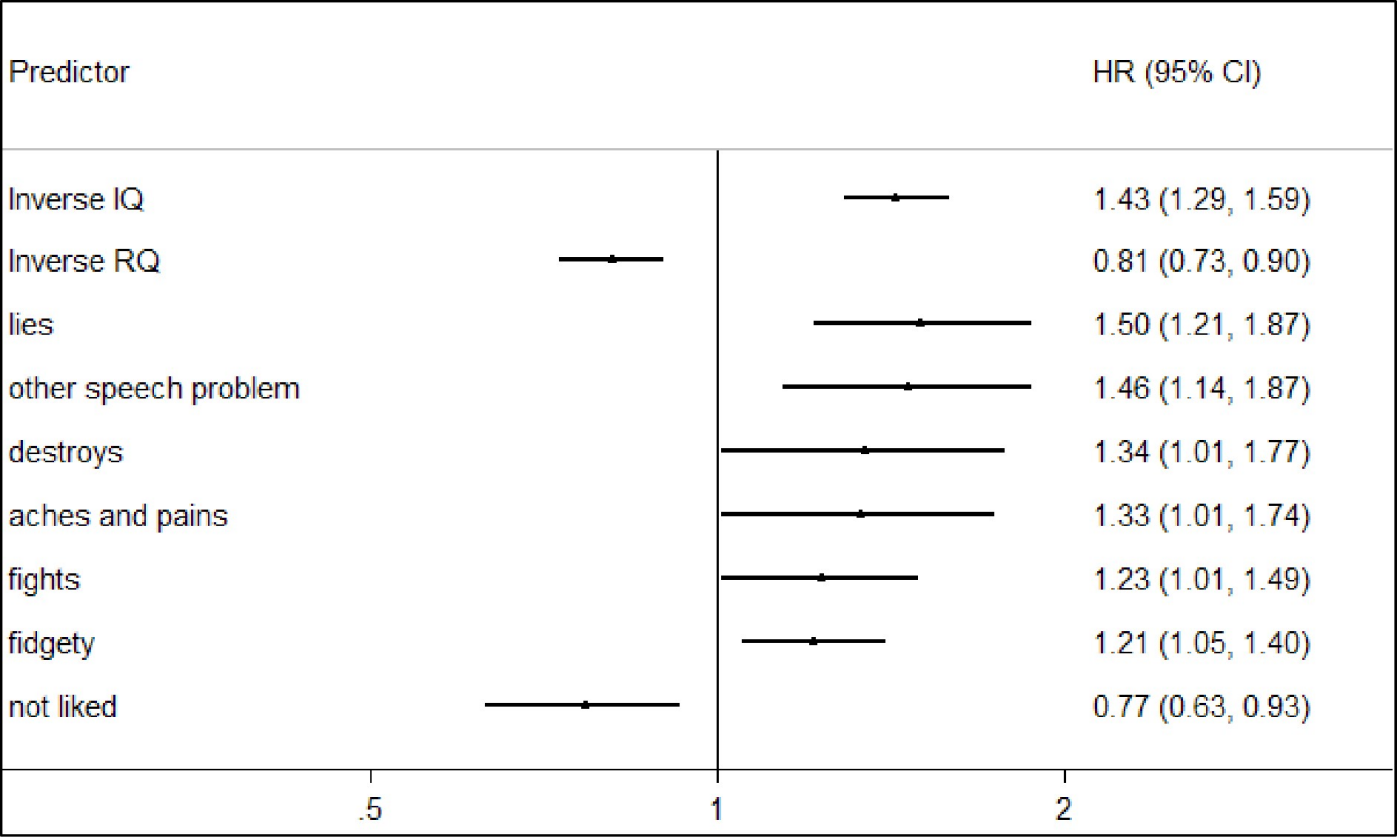
Selected model from analysis of Rutter B items and cognitive measures (n = 11,241).

The individual Rutter B behaviours were entered simultaneously together with IQ and RQ into an adjusted survival analysis model derived by backwards elimination. The results are displayed in Fig 2. In this adjusted model, gender, IQ and RQ were retained along with seven Rutter B behaviours. Six of the Rutter B behaviours showed a significant independent positive association with premature mortality (lies, other speech problem, destroys, aches and pains, fights, fidgety), whilst not being liked changed direction compared with the univariate analysis. Of the Rutter B behaviours that predicted a greater risk of death, hazard ratios ranged from 1.21 to 1.50. The magnitude of the association between IQ and premature mortality was increased within this model compared with the univariate analysis. Lower IQ remained a significant predictor of premature mortality, with a one standard deviation decrease in IQ associated with a 43% greater risk of death (HR: 1.43, 95% CI: 1.29,1.59, p<0.001). The effect of RQ also changed direction from the univariate analysis when entered into this model (HR: 0.81 95% CI: 0.73,0.90, p <0.001). This was as a result of adjusting for IQ which it is highly correlated with RQ, as the HR was also less than one when RQ and IQ were entered into a model without the Rutter B behaviours (RQ HR:0.82, 95% CI: 0.74,0.91, p<0.001; IQ HR 1.52, 95% CI:1.37,1.69, p<0.001).

## Discussion

### Findings

Our findings demonstrated an association between a wide range of neurodevelopmental markers, including reduced intellectual functioning, and greater risk of all-cause premature mortality. The hazard ratios of between 1.24 and 2.28 are of similar magnitude to results from other studies of childhood behaviour and premature mortality e.g. Maughan et al report hazard ratios of 1.17 in men and 1.16 in women [3]. To highlight the significance of these results they can be compared to studies of smoking and all-cause mortality (HR 1.3–2.7) [31, 32]. The most commonly reported markers were attention problems (30.1%), fidgetiness (22.7%) (both core symptoms of ADHD diagnosis) and nail biting (19.8%) which is associated with a range of emotional and behavioural difficulties [33]. In the adjusted model six individual neurodevelopmental markers (lies, speech problems, destroys, fights, aches and pains, fidgety) were positively associated with premature mortality. These varied markers index several problem domains, each adding significantly to the prediction of premature mortality by between 21% and 50%. These domains are: problems with hyperactivity (fidgety), speech problems (common in autism), conduct problems (destroys, fights, lie*s*), and complaining of aches and pains (considered a proxy marker of anxiety in children). This shows, for the first time, that childhood neurodevelopmental markers in any domain are associated with higher risk of premature mortality. Each standard deviation of lower IQ was associated with a more than 40% greater risk of premature mortality. This finding is consistent with previous work in this cohort and in other studies (Calvin et al, 2011), but our study is novel in demonstrating that individual neurodevelopmental markers predict premature mortality independently of the known association between premature mortality and IQ.

It is difficult to account for the result that “not being liked” reduced the risk of premature death in the adjusted model, especially as poor peer relations and conduct problems (which greatly increase premature mortality) often go together [34]. This finding needs to be corroborated and investigated further in other studies.

When controlling for IQ, higher Reading Quotient was associated with a greater risk of premature mortality. A discrepancy between advanced reading ability and poor comprehension are features of “hyperlexia” which is strongly associated with autism [35]. This finding would need replication and further investigation.

There is already a significant body of research demonstrating premature mortality associated with individual neurodevelopmental diagnoses [1–3, 5] but many children in the general population are likely to have one or two of these neurodevelopmental markers and most will not attract a neurodevelopmental diagnosis. This should alert us to the importance of symptom load in child neurodevelopment. In other fields, such as cardiovascular science, risk scores that calculate global risk across a range of health parameters (e.g. blood pressure, weight and serum lipid profile) have proven invaluable in identifying people most at need of early intervention to prevent the onset of disease [36]. Our findings could open the door to new consideration of child mental public health with a focus on risk indicators considered individually and cumulatively with the aim of reducing the risk of broad health outcomes at a population level. At a clinical level, these findings stress the importance of the ESSENCE approach, i.e. holistic assessment and treatment of children presenting with developmental difficulties in order to improve health across the lifespan.

### Strengths and weaknesses

A major strength of this study is the examination of the data from an ESSENCE perspective utilising individual Rutter B behaviours rather than the traditional method of simply calculating a total Rutter problem score or using single diagnostic categories. The study had a large sample size (n = 11,241) with 92.5% of the cohort available for complete case analysis. There has been a sufficiently long follow-up period of fifty years. The study includes those across a spectrum of cognitive abilities including those with intellectual disabilities attending specialist schools in addition to those in mainstream primary schools. Linkage to national records is a particular strength as the study was able to include those that may traditionally be lost to follow up (e.g. those with lower IQ) ensuring the study population was representative of the general population of the time. We only used the teacher reported scale and, while parent and teacher report can differ slightly in the way they describe a child’s problems, the Rutter B (teacher) scale has good psychometric properties even compared to the parent version [29]. It possible that including a parent-report version (which asks slightly different questions) might have added further insights about risks for premature mortality but the numbers with parent-report questionnaires was too low for these kinds of analyses. It would also have been interesting to examine the extent to which these teacher-reported neurodevelopmental markers resulted in referrals for clinical assessments and this could be a focus of future research using data linkage.

### Implications

This study highlights the importance of considering childhood neurodevelopmental markers, including IQ, across all domains, including symptoms that are “sub-threshold” for individual diagnoses, given the contribution of these to premature mortality. ESSENCE is relatively recently described, and our study provides direct evidence to support this approach as well as the need for consideration of a broad range of neurodevelopmental markers in assessment and treatment of children with neurodevelopmental presentations.

### Future research

Further investigation is needed to better understand the mechanisms linking childhood neurodevelopmental markers with adult health outcomes that are responsible for excess premature deaths. Important next steps will be to investigate how these neurodevelopmental markers cluster, how they change in their expression and impact across childhood, through adolescence and into adulthood and how they relate–together and in clusters–to disease processes. A better understanding of the underlying causes of premature mortality may enable the development of preventative approaches that are relevant to children and adults with ESSENCE symptoms.

## Conclusion

This study of 1,167 premature deaths in a general population cohort of 11,241 participants has demonstrated that neurodevelopmental markers relating to the major domains of child psychopathology, including reduced intellectual functioning in childhood, substantially increase the risk of premature mortality.

## Supporting information

S1 Table Rutter B Items and mortality, unadjusted and adjusted for gender. (DOCX)

S1 Appendix (DOCX)
