# Supplementary material for: Childhood neurodevelopmental markers and risk of premature mortality: Follow-up to age 60–65 years in the Aberdeen Children of the 1950s study
Source: PLoS One. 2021 Aug 18;16(8):e0255649. doi: 10.1371/journal.pone.0255649 (PMC8372930; doi:10.1371/journal.pone.0255649)
Supplement: S1 Table — (DOCX) [file pone.0255649.s001.docx]

**S1 Table. Rutter B Items and mortality, unadjusted and adjusted for gender.**

|  | | | | | | | | | |
| --- | --- | --- | --- | --- | --- | --- | --- | --- | --- |
|  | Unadjusted | | | |  | Adjusted for gender | | | |
|  | HR | LCL | UCL | P |  | HR | LCL | UCL | P |
| restless | 1.40 | 1.21 | 1.63 | 0.000 |  | 1.29 | 1.11 | 1.50 | 0.001 |
| truants | 2.16 | 1.57 | 2.98 | 0.000 |  | 1.94 | 1.41 | 2.67 | 0.000 |
| fidgety | 1.43 | 1.25 | 1.63 | 0.000 |  | 1.34 | 1.17 | 1.53 | 0.000 |
| destroys | 1.80 | 1.38 | 2.36 | 0.000 |  | 1.61 | 1.23 | 2.11 | 0.001 |
| fights | 1.60 | 1.36 | 1.90 | 0.000 |  | 1.50 | 1.27 | 1.78 | 0.000 |
| Not liked | 1.40 | 1.17 | 1.67 | 0.000 |  | 1.37 | 1.14 | 1.63 | 0.001 |
| worries | 0.98 | 0.83 | 1.15 | 0.801 |  | 0.99 | 0.84 | 1.17 | 0.943 |
| solitary | 1.11 | 0.93 | 1.33 | 0.241 |  | 1.06 | 0.89 | 1.26 | 0.521 |
| irritable | 1.61 | 1.34 | 1.94 | 0.000 |  | 1.50 | 1.25 | 1.81 | 0.000 |
| unhappy | 1.21 | 0.98 | 1.49 | 0.073 |  | 1.20 | 0.97 | 1.48 | 0.086 |
| tics | 1.22 | 0.90 | 1.64 | 0.197 |  | 1.07 | 0.79 | 1.44 | 0.681 |
| sucks | 1.13 | 0.87 | 1.46 | 0.367 |  | 1.17 | 0.91 | 1.51 | 0.228 |
| nails | 1.21 | 1.05 | 1.40 | 0.010 |  | 1.23 | 1.07 | 1.43 | 0.005 |
| schl absences | 1.47 | 1.17 | 1.84 | 0.001 |  | 1.46 | 1.16 | 1.83 | 0.001 |
| disobedient | 1.72 | 1.46 | 2.02 | 0.000 |  | 1.56 | 1.33 | 1.84 | 0.000 |
| attention | 1.50 | 1.33 | 1.70 | 0.000 |  | 1.45 | 1.28 | 1.64 | 0.000 |
| afraid new | 1.02 | 0.86 | 1.21 | 0.838 |  | 1.06 | 0.89 | 1.25 | 0.534 |
| fussy | 0.85 | 0.64 | 1.13 | 0.260 |  | 0.91 | 0.69 | 1.20 | 0.500 |
| lies | 2.02 | 1.69 | 2.43 | 0.000 |  | 1.86 | 1.55 | 2.23 | 0.000 |
| steals | 2.46 | 1.84 | 3.27 | 0.000 |  | 2.21 | 1.66 | 2.95 | 0.000 |
| wet/soil | 1.00 | 0.58 | 1.72 | 0.988 |  | 0.93 | 0.54 | 1.61 | 0.793 |
| aches | 1.33 | 0.99 | 1.78 | 0.062 |  | 1.38 | 1.03 | 1.86 | 0.033 |
| tears/schlrefusal | 0.69 | 0.26 | 1.84 | 0.458 |  | 0.68 | 0.26 | 1.82 | 0.446 |
| stutter | 1.43 | 1.00 | 2.05 | 0.049 |  | 1.24 | 0.87 | 1.77 | 0.243 |
| other/speech | 1.62 | 1.26 | 2.08 | 0.000 |  | 1.45 | 1.13 | 1.87 | 0.004 |
| bullies | 1.58 | 1.25 | 2.00 | 0.000 |  | 1.43 | 1.13 | 1.81 | 0.003 |
