# Supplementary material for: Childhood neurodevelopmental markers and risk of premature mortality: Follow-up to age 60–65 years in the Aberdeen Children of the 1950s study
Source: PLoS One. 2021 Aug 18;16(8):e0255649. doi: 10.1371/journal.pone.0255649 (PMC8372930; doi:10.1371/journal.pone.0255649)
Supplement: S1 Appendix — (DOCX) [file pone.0255649.s002.docx]

**S1 Appendix**

| Rutter B question wording | Paper short form |
| --- | --- |
| Very restless. Often running about or jumping up and down. Hardly ever still | *restless* |
| Truants from school | *truants* |
| Squirmy, fidgety child | *fidgety* |
| Often destroys own or others’ belongings | *destroys* |
| Frequently fights with other children | *fights* |
| Not much liked by other children | *not liked* |
| Often worried, worries about many things | *worries* |
| Tends to do things on his own – rather solitary | *solitary* |
| Irritable. Is quick to ‘fly off the handle’ | *irritable* |
| Often appears miserable, unhappy, tearful or distressed | *unhappy* |
| Has twitches, mannerisms or tics of the face or body | *tics* |
| Frequently sucks thumb or finger | *sucks fingers* |
| Frequently bites nails or fingers | *bites nails* |
| Tends to be absent from school for trivial reasons | *school absences* |
| Is often disobedient | *disobedient* |
| Has poor concentration or short attention span | *attention* |
| Tends to be fearful or afraid of things or new situation | *afraid of new things* |
| Fussy or over-particular child | *fussy* |
| Often tells lies | *lies* |
| Has stolen things on one or more occaions | *steals* |
| Has wet or soiled self at school this year | *wets or soils* |
| Often complains of pains or aches | *aches and pains* |
| Has had tears on arrival at school or has refused to come into the building this year | *tears/school refusal* |
| Has a stutter or stammer | *stutters* |
| Has other speech difficulty | *other speech problem* |
| Bullies other children | *bullies* |
